# Human IgM–expressing memory B cells

**DOI:** 10.3389/fimmu.2023.1308378

**Published:** 2023-12-08

**Authors:** Bettina Budeus, Artur Kibler, Ralf Küppers

**Affiliations:** Institute of Cell Biology (Cancer Research), Medical Faculty, University of Duisburg–Essen, Essen, Germany

**Keywords:** B cells, CD27, class switch recombination, germinal center, IgD, immunoglobulin V genes, marginal zone, somatic hypermutation

## Abstract

A hallmark of T cell dependent (TD) humoral immune responses is the generation of long–lived memory B cells. The generation of these cells occurs primarily in the germinal center (GC) reaction, where antigen–activated B cells undergo affinity maturation as a major consequence of the combined processes of proliferation, somatic hypermutation of their immunoglobulin V (IgV) region genes, and selection for improved affinity of their B–cell antigen receptors. As many B cells also undergo class–switching to IgG or IgA in these TD responses, there was traditionally a focus on class–switched memory B cells in both murine and human studies on memory B cells. However, it has become clear that there is also a large subset of IgM–expressing memory B cells, which have important phenotypic and functional similarities but also differences to class–switched memory B cells. There is an ongoing discussion about the origin of distinct subsets of human IgM^+^ B cells with somatically mutated IgV genes. We argue here that the vast majority of human IgM–expressing B cells with somatically mutated IgV genes in adults is indeed derived from GC reactions, even though a generation of some mostly lowly mutated IgM^+^ B cells from other differentiation pathways, mainly in early life, may exist.

## Introduction

A major strength of the human immune system is that it is not only capable of combating infectious agents, such as viruses and bacteria, during a first encounter with these pathogenic microorganisms, but that it also establishes immunological memory. This immunological memory prepares our immune system for a faster and improved response upon re–encounter with the same or a related infectious agent. Immunological memory is a feature of the lymphocytes of the adaptive immune system, i.e., B cells and T cells. B lymphocytes recognize foreign antigens through their B–cell antigen receptor (BCR), which can be secreted as soluble antibody if B cells differentiate into plasmablasts or plasma cells. Memory within the B–cell system is largely, if not exclusively, generated during T cell dependent (TD) immune responses, which include the germinal center (GC) reaction. In the GC reaction, two types of long–lived descendants of antigen–activated B cells are generated, namely memory B cells and plasma cells (1). Whereas plasma cells have no further proliferative potential and cannot modify the fine–specificity of the antibody molecules they produce, memory B cells are more flexible in their behavior upon re–encounter of the antigen for which their BCR is specific. After reactivation they can either proliferate and then become plasma cells, or they may reenter a GC reaction for further improvement of the affinity of their BCR or its adaptation to a modified antigen (2). Distinct subsets of human memory B cells have been identified, and a major distinction is between IgM–expressing and class–switched memory B cells (2). However, there is still an ongoing discussion about the origin, heterogeneity, and specific functions of IgM–expressing memory B cells in humans. This review is focused on discussing the current knowledge and ideas about human IgM^+^ memory B cells.

## B–cell development

**Figure 1:**
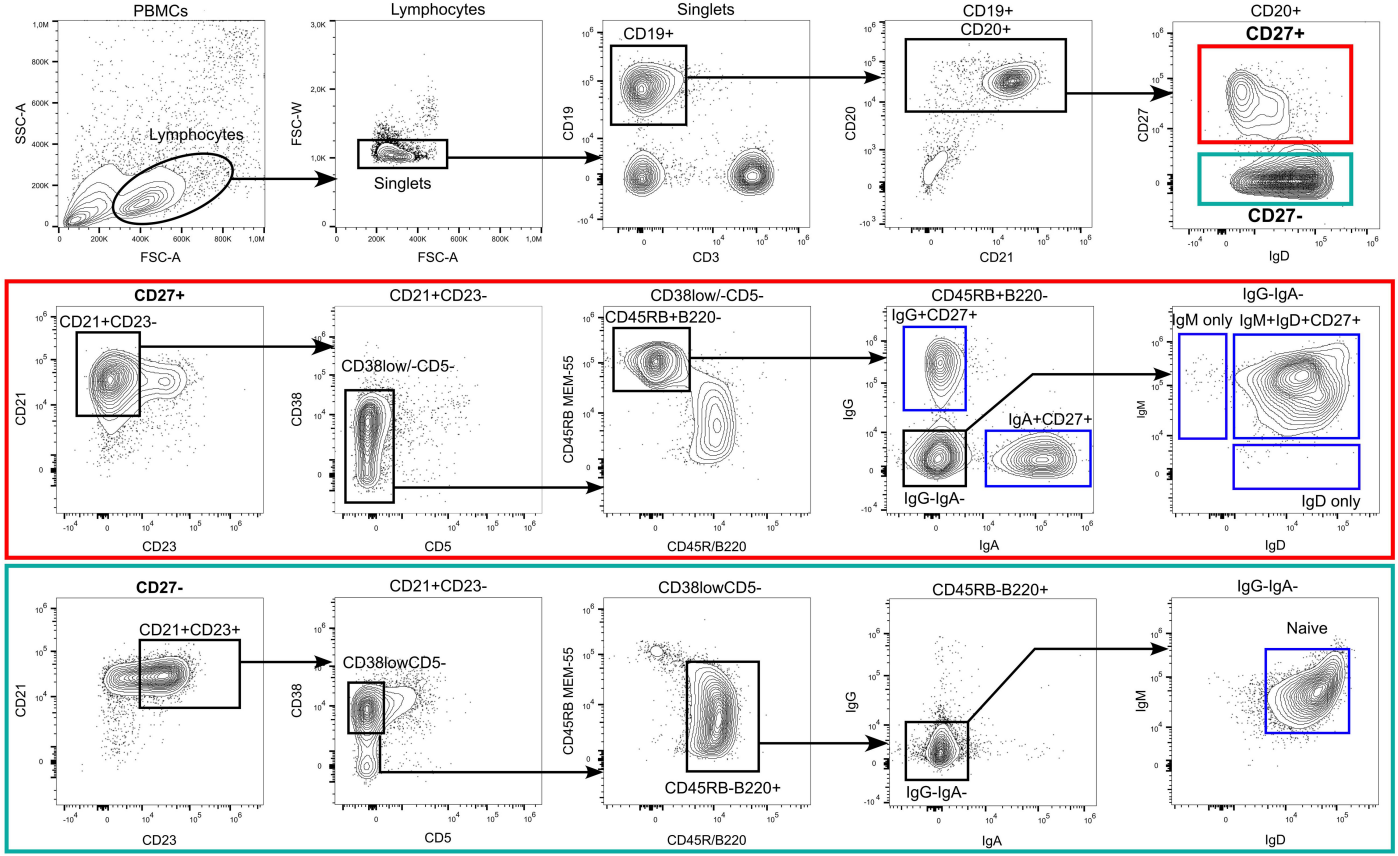
Flow cytometric identification of human B–cell subsets. Among peripheral blood–derived mononuclear cells (PBMC), most mature B cells can be identified by the expression of the pan B cell markers CD19 and/or CD20. CD19 also stains plasma blasts and most plasma cells, which are mostly CD20^low^CD21^low^ and surface BCR^low^ in peripheral blood. The choice of one or more of these molecules, to include into the FACS panel depends on the B–cell subsets of interest, expression of additional B–cell hallmark molecules, such as the heavy or light chains of the BCR, and the configuration of the flow cytometer. Exclusion of non–B–cells is often recommendable, as is depicted in the plot for the CD19 and CD3 staining. In addition, markers such as CD56 (natural killer cells), CD14, and CD16 (monocytes and granulocytes) can be co–stained using the same fluorescent dye as CD3 (creating a dump channel; not shown here). Mature naive and memory B cells can be distinguished by the differential expression of CD23, CD27, CD45RB (clone MEM–55), CD45R/B220 (clone RA3–6B2) and the expression of class–switched BCR isotypes such as IgG and IgA. Mature naive B cells express the phenotype CD23^pos^CD27^neg^CD45RB^neg^B220^pos^CD38^low^CD5^neg^IgG^neg^IgA^neg^IgD^pos^IgM^pos^. However, not all of these markers are mandatory for a routine identification of naive B cells, for which we recommend stainings at least for CD19 or CD20, IgD, CD23, and CD27. Most memory B cells express the phenotype CD23^neg^CD27^pos^CD45RB^pos^B220^neg^ and can be distinguished by their expression of surface BCR isotypes (IgM/IgG/IgA/IgE). In addition, a small fraction of CD27^neg^IgA^pos^ and CD27^neg^IgG^pos^ memory B cells exist in human blood and lymphoid tissues. A core panel to define class–switched memory B cells should include CD19 or CD20, IgG and/or IgA, and CD27. Please note that the frequently used marker constellation IgD^–^CD27^+^ also includes a small subset of IgM–only memory B cells. In addition, small subsets of CD27^neg^IgA^pos^ and CD27^neg^IgG^pos^ memory B cells exist in human blood and lymphoid tissues. CD24 and CD10 can support the differentiation of mature B cells from transitional or immature B cells (transitional B cells express CD24^high^CD10^high^ in addition to CD38^pos^CD5^pos^), which is particularly important when analyzing bone marrow, and from GC B cells (CD24^low^CD10^high^ in addition to CD38^pos^), which is of interest when analyzing secondary lymphoid organs. Additional subsets that were not pointed out in the gating strategy include atypical B cells (CD21^low^Tbet^pos^CD183^pos^CD11c^pos^) and CD5 mature naive B cells (CD27^neg^CD23^pos^CD38^low^CD5^pos^). The CD5 mature B cells in adults are phenotypically and functionally similar to CD5^neg^ naive B cells, however, in neonates and children these two populations differ substantially (8).

Human B lymphocytes are generated from common lymphoid progenitors in a stepwise differentiation process that takes place in postnatal life in the bone marrow (3). The main task of early B–cell development is to equip each B cell with a BCR. This is mediated through several somatic recombination events which assemble the variable parts of the immunoglobulin (Ig) heavy and light chain genes, mediated by the enzymes recombination activating gene (RAG)1 and RAG2 (4). For the heavy chain, three gene segments have to be assembled, the variable (V), diversity (D), and joining (J) genes, whereas the kappa and lambda light chain V region genes are composed of a V and a J gene (5). The estimated potential diversity of the human mature BCR repertoire is 10^12^ to 10^18^ unique rearrangements (6). Several factors contribute to this enormous diversity of the BCR repertoire: First, there is a substantial number of different germline IGHV, IGHD and IGHJ genes that can be recombined to encode the Ig heavy chain V region, and numerous IgV and IgJ genes can be assembled to generate the kappa and lambda light chain V regions in a process called V(D)J–recombination (5, 7). Second, the random integration and removal of nucleotides at the junctions of the rearranging genes massively increases diversity (7). Third, multiple combinations of rearranged Ig heavy and light chains can pair to form a stable BCR. These mechanisms lead to the generation of naive, mature B cells, which are equipped with a unique BCR. The unique nucleotide sequence [in particular that of the complementarity determining region (CDR) III of the heavy chain, which encompasses the IGHV–IGHD–IGHJ joining site] of the BCR can be used as a clonal marker to trace the progeny of this single cell during immune responses. Mature B cells exit the bone marrow and circulate through the body in search for foreign antigens. Naive B cells coexpress the BCR as IgM and IgD molecules, which is mediated through differential splicing of the primary heavy chain transcripts. These cells can be identified in flow cytometric studies through staining with a combination of antibodies (see **Figure 1**).

## Germinal center reaction

TD immune responses are initiated when antigen–activated B cells meet antigen–specific T helper (TH) cells in the T–cell region or the T–B region border of secondary lymphoid organs, such as lymph nodes. Antigen– and TH cell–stimulated B cells then proliferate, building a primary focus (9). Some of the B cells differentiate into short lived plasma cells, and studies in the mouse showed that they can also differentiate into TD memory B cells with unmutated IgV genes without participating in the GC reaction (10). Other B cells of the primary focus reaction migrate into neighboring B–cell follicles. In the follicle, the activated B cells undergo massive proliferation, thereby establishing a histological structure called GC (1). The GC reaction is the major process that generates memory B cells with high–affinity BCRs and plasma cells secreting high affinity antibodies in a TD fashion. The main players besides the antigen–activated GC B cells are T follicular helper (TFH) cells and follicular dendritic cells (FDCs). B cells in the GC undergo regulated cycles of proliferation, IgV gene mutation, and selection for improved affinity of their BCR. A GC can be histologically subdivided into the dark zone and the light zone (1). GC B cells in the dark zone (centroblasts) vigorously proliferate and undergo somatic hypermutation (SHM) of their Ig heavy and light chain V region genes (11, 12). SHM is initiated by activation–induced cytidine deaminase (AID), which deaminates C bases, giving rise to U bases (13). Error–prone repair of the U bases then leads to the generation of somatic mutations. After a few cell divisions, centroblasts cease proliferation and SHM and move to the light zone of the GC where they become resting centrocytes (1). Centrocytes are then selected for improved binding of their BCR to the cognate antigen, which involves presentation of antigenic peptides loaded on major histocompatibility complex II molecules to the T–cell receptor of TFH cells (1, 14). The selection and differentiation process of GC B cells involves further receptor–ligand interactions, such as binding of CD40 on GC B cells to CD40L on TFH cells, and secreted factors, such as interleukins 4 and 21 (1). The vast majority of GC B cells will not acquire affinity–increasing mutations, and these cells rapidly undergo apoptosis (1). GC B cells typically undergo multiple rounds of proliferation, SHM and selection, leading to a stepwise improvement of BCR affinity. TFH cells also direct the further differentiation of GC B cells into either memory B cells or plasma cells (1). Memory B cells are often generated earlier in a GC reaction than plasma cells, as the antibodies produced by plasma cells often have a higher somatic mutation load and a higher affinity for the antigen than the BCR of memory B cells (15).

A further Ig gene remodeling process is class switch recombination (CSR). This process is, like SHM, mediated by AID, which causes DNA double strand breaks in the switch regions upstream of the IGH constant region genes (13). CSR leads to the exchange of the constant part of the Ig heavy chain, so that instead of co–expression of IgM and IgD now immunoglobulins of the classes IgG, IgA and IgE with distinct effector functions can be produced. Whereas initial findings point to a predominant activity of CSR in the light zone of the GC, recent findings in mice and humans indicate that many antigen-activated B cells in TD immune responses undergo CSR already prior to entering the GC (2, 16, 17).

A cell surface receptor with high relevance for the further discussion is the tumor necrosis factor receptor (TNFR) superfamily member CD27. CD27 is not expressed by naive B cells, is upregulated on GC B cells and remains expressed on most memory B cells (18–20). It is highly expressed on post–GC plasma cells (20, 21). CD27 has been proposed as a general memory B–cell marker in humans (18, 19).

## Human memory B–cell subsets

**Figure 2:**
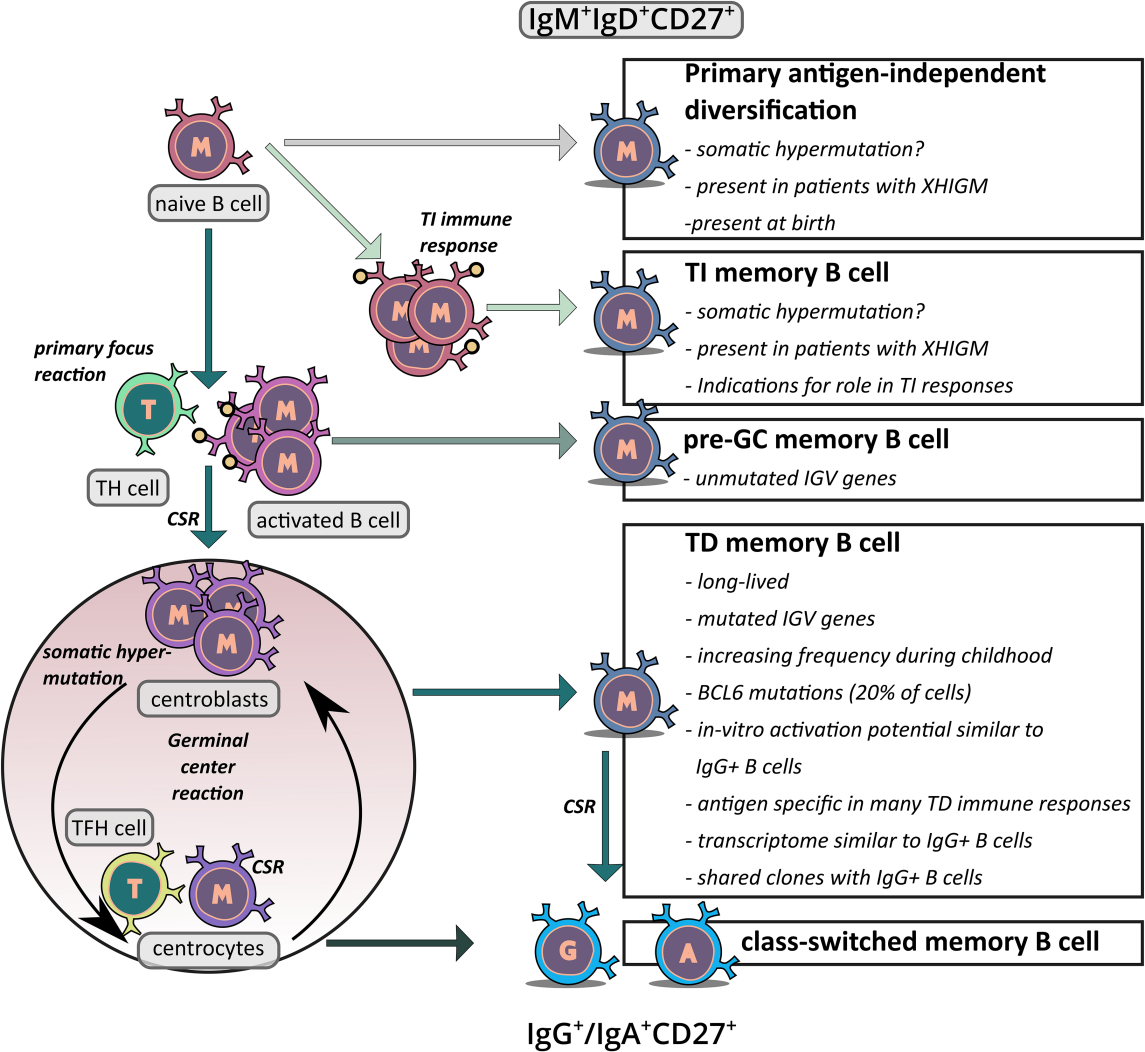
Schematic overview of the proposed generation pathways of IgM^+^IgD^+^CD27^+^ memory B cells. The figure depicts the potential origins of IgM^+^IgD^+^CD27^+^ B cells. First, there is indication that at least in young children, a subset of these cells may be derived from an antigen–independent primary diversification process in which the cells acquire a low level of somatic mutations. Second, it has been proposed that IgM^+^IgD^+^CD27^+^ B cells are derived from TI immune responses and hence represent memory B cells which were generated without T cell help. Third, in TD immune responses some memory B cells may be generated in the primary focus reaction when cognate B and T cells have their first interaction, before SHM is activated. This could be the origin of IgM^+^IgD^+^CD27^+^ B cells without IGV gene mutations. Fourth, at least in adults the vast majority of IgM^+^IgD^+^CD27^+^ B cells presumably derives from TD GC responses, which also give rise the classical class–switched memory B cells. Key arguments for and unresolved issues of the differentiation pathways discussed are indicated in the boxes.

Historically, memory B cells were identified by the expression of class–switched BCR, in particular IgG and IgA. This is because CSR was considered as a main hallmark of B cells that underwent a GC reaction and became memory B cells, and as surface IgG and IgA can be easily used to detect and isolate these cells. However, it is now clear that also IgM–expressing memory B cells exist, and further subsets of memory B cells have been identified (**Figure 2**). Before discussing IgM–expressing memory B cells – the main topic of this review – we provide a short overview over the other main subsets of human memory B cells.

### IgG memory B cells

In humans, there are four functional IgG isotypes (IgG1, IgG2, IgG3, and IgG4). About 80% of IgG memory B cells express the CD27 marker, and IgG^+^ B cells typically account for about 10–15% of B cells in the peripheral blood of healthy adults (19, 22–24) (**Figure 1**, **Table 1**). IgG1 and IgG2 are most frequently used by IgG memory B cells, whereas IgG4^+^ B cells are very rare (24–26). Nearly all IgG^+^ memory B cells carry somatically mutated IgV genes, with average mutation frequencies of 5–7% (19, 25, 27, 28). As a hallmark of memory B cells, IgG^+^ memory B cells can be faster and stronger activated than naive B cells (29–37). In vitro, such reactivated IgG memory B cells have a propensity to differentiate into plasmablasts and plasma cells, instead of acquiring a GC program (29).

**Table 1:** Frequencies of human B–cell subsets in peripheral blood in different age groups.

B–cell subset	Phenotype	0–2 y N = 5	3–9 y N = 3	10–19 y N = 18	20–59 y N = 50	≥ 60 y N = 18
**CD27–negative**		**92 (74, 92)**	**87 (76, 89)**	**81 (75, 85)**	**75 (63, 79)**	**76 (65, 83)**
Transitional	CD27^–^CD23^+^CD38^+^IgM^+^IgD^+^CD5^+^	9 (7, 11)	18 (18, 18)	3 (3, 3)	3 (3, 4)	2 (1, 2)
Naive	CD27^–^CD23^+^CD38^low^IgD^+^IgM^+^CD5^–^	56 (36, 85)	76 (59, 82)	58 (50, 74)	45 (36, 53)	44 (37, 53)
Mature CD5^+^	CD27^–^CD23^+^CD38^low^IgD^+^IgM^+^CD5^+^	17 (16, 18)	19 (19, 19)	6 (6, 6)	5 (4, 8)	8 (7, 8)
**CD27^+^ memory**		**8 (8, 15)**	**13 (11, 15)**	**19 (14, 25)**	**25 (21, 36)**	**25 (19, 35)**
IgM^+^IgD^+^ memory	CD27^+^IgM^+^IgD^+^	8 (5, 14)	8 (6, 9)	9 (6, 10)	10 (8, 16)	9 (6, 14)
IgG/IgA memory	CD27^+^IgM^–^IgD^–^ or CD27^+^IgG/IgA^+^	1 (1, 1)	5 (4, 5)	7 (4, 11)	11 (7, 13)	9 (5, 12)

Shown are frequencies among all B cells of healthy individuals as median of the donors (with lower quartile and upper quartile in brackets) in %, distinguished into CD27–negative and CD27–positive B cells. The phenotype of these cells is specified. Please note that as quartiles for groups with 5 up to 50 donors are given, the sum does not necessarily add up to 100%, or the frequency of the main cell type category. Moreover, also because of stringent gating for subsets, the sum of the frequencies of the subsets is typically lower than the frequencies of the main category. N denotes the number of blood donors per age group. y, years–old.

Data in bold are those for the B-cell main divisions.

### IgA memory B cells

IgA^+^ memory B cells account for about 10% of B cells in the peripheral blood of healthy adults (24). Similar to IgG memory B cells they mostly express CD27, and the vast majority of CD27^+^IgA^+^ memory B cells carry somatically mutated IgV genes, at levels similar to IgG memory B cells (28, 38). IgA^+^ memory B cells are mostly generated in the intestine and other mucosal tissues. The most important IgA–inductive sites in the gut are Peyer’s patches (PP), clusters of follicles where IgA^+^ B cells are generated (39, 40). The second structure responsible for IgA B–cell development are isolated lymphoid follicles, which are smaller than PPs and often contain only one follicle (39). In mice there are comprehensive data that a fraction of IgA memory B cells and plasma cells are generated in T–cell independent (TI) immune responses (41, 42). However, in humans the high level of somatic IgV gene mutations seen in the vast majority of IgA^+^CD27^+^ memory B cells and plasma cells argues that these cells are mainly generated in GC reactions of TD immune responses (38, 43, 44).

### IgD–only B cells

A very rare (<1% of B cells in peripheral blood) and peculiar subset of memory B cells is characterized by expression of IgD in the complete absence of IgM, so–called IgD–only B cells (19, 45). Whereas mature naive B cells and IgM–expressing memory B cells co–express IgM and IgD, and may finetune their relative expression depending on the particular differentiation and activation stage (46), IgD–only B cells have undergone an atypical CSR event involving the Sμ region and a pentamer–rich σ/δ region upstream of Cδ (47), causing deletion of all Cμ exons, so that these cells cannot express any IgM anymore. IgD–only B cells were first identified as the malignant clones in some patients with multiple myeloma (48) and hairy cell leukemia (49). IgD–only memory B cells derive from IgD–only GC B cells, which are mainly found in tonsils, and these GC B cells also give rise to IgD–secreting plasma cells (47, 50). IgD–only B cells show a very high IgV gene mutation frequency, a highly biased usage of λ light chains, and a preferred usage of the *IGHV3–30* gene (19, 50, 51). These features indicate that those cells derive from a peculiar, likely super–antigen–driven, GC reaction (19, 50, 51).

### IgE memory B cells

IgE^+^ memory B cells are very rare in the human body and account for about 0.1% of peripheral blood B cells in adults (52). IgE has a physiological role in immune responses against parasites, but it has also a main role in allergic reactions, as IgE antibodies are capable of activating mast cells (53). In mice, IgE^+^ GC B cells are produced early during the immune response and then quickly disappear (54–56). It is not clear if this is also the case in humans, but the current concept in mice and humans is that the main source of IgE–producing plasma cells is not the very small pool of IgE^+^ memory B cells, but IgG memory B cells that upon particular stimuli undergo secondary CSR to IgE and then differentiate into IgE–secreting plasma cells (55, 57).

### CD27–negative memory B cells

As already briefly mentioned, CD27, a cell surface receptor contributing to B–cell differentiation, expansion, antibody production, and B cell–T cell–interaction (via its ligand CD70) is often used to distinguish memory B cells from naive B cells, as naive B cells lack CD27 expression and most memory B cells express CD27 on their surface (18, 19, 58, 59). CD27^+^ memory B cells are IgV gene mutated and often found as classical post–GC class–switched B cells (60, 61). But not all cells without CD27 are naive B cells. CD27–negative memory B cells (recognized as such because of their mutated IgV genes) represent about 5% of all B cells in the periphery in healthy adults and are already present at low frequency at birth (22, 62–64). It was proposed that such CD27^–^ and also CD27^dull^ memory B cells may be a form of precursor for CD27^+^ memory B cells (65), as there exists a high degree of similarity between the BCR repertoires of CD27^–^ and CD27^+^ memory B cells, and they are frequently members of common clones (28). Moreover, IgG^+^CD27^–^ memory B cells express a lower level of somatic IgV gene mutations compared to their CD27–positive counterpart (22) and single cell targeted DNA methylation analysis showed a progress of cellular differentiation with increase of CD27 expression (66), which may further support this theory.

### CD21^low^T–bet^high^ B cells

Typically, all mature B cells express the complement receptor 2 (CR2, also termed CD21). A distinct subset of CD21^low^ mature B cells was first described in immune deficiency conditions, such as in patients with HIV infection (67, 68), but were later also identified in other chronic viral and parasitic infections, and in patients with autoimmune diseases (69, 70). In healthy individuals, these cells are very rare in peripheral blood. A corresponding population has been found at increased frequency in old mice and were hence termed age–associated B cells in these animals (71). Most of the CD21^low^ B cells express the transcription factor T–bet at high level, which was originally described as a master regulator of commitment of CD4^+^ T cells to the T helper 1 (TH1) cell lineage (72). The CD21^low^T–bet^high^ B cells are often also positive for CD11c and FCRL4 (69, 70). As different combinations of these markers were used in the various studies of CD21^low^ and/or T–bet^high^ B cells, which are often also called atypical B cells, it is not clear whether this is a homogenous B–cell subset or whether in distinct disease settings different subtypes of these cells with distinct marker combinations exist. Indeed, in the various diseases, the proportions of CD27^+^ and class–switched B cells among these cells varies considerably (69, 70). Importantly, however, CD21^low^T–bet^high^ B cells show strong indications of being memory B cells. Most of these cells carry somatically mutated IgV genes at levels similar to classical human memory B cells, they need T–cell help for their generation, they express pathogen–specific BCRs in chronic infections, they are often CD27– and/or IgG–expressing, and they can include common clones with classical CD27^+^CD21^+^ memory B cells (69, 70, 73, 74). Initially, CD21^low^T–bet^high^ B cells were considered as exhausted B cells, as they responded poorly to BCR cross–linking. However, later studies showed that if BCR triggering is combined with other stimuli, e.g., specific cytokines and/or Toll–like receptor triggering, these cells responded similarly fast and strong as classical memory B cells (69, 70).

## Potential origins of somatically mutated human IgM^+^ B cells

It is well established that most if not all class–switched memory B cells in humans are generated during GC reactions in TD immune responses. However, the generation of IgM^+^IgD^+^ B cells with somatically mutated IgV genes is still debated. Three different possible origins of these cells were proposed: the “classical” GC–derived pathway, an antigen independent primary diversification developmental pathway, and a derivation from TI immune responses (61) (**Figure 2**). The possible GC derivation of these cells will be discussed in detail in the following chapter. Here, we address the two alternative developmental pathways for the generation of somatically mutated IgM^+^IgD^+^ B cells.

The first indication for a GC–independent origin of human somatically mutated IgM^+^IgD^+^CD27^+^ B cells derived from the observation that such cells can be identified in patients affected by the X–linked hyper–IgM syndrome type 1 (HIGM1) (75). In these patients the *CD40L* gene is defective, which is thought to be essential for GC reactions, and class–switched memory B cells are indeed absent in these patients (75). As IgM^+^IgD^+^CD27^+^ B cells appear in young children before class–switched memory B cells are detectable, and develop early on a diversified BCR repertoire, this may indicate a GC and even immune response independent generation of these cells (76). A primary, antigen–independent BCR diversification through SHM is indeed known from some animal species (77). In further support of their antigen–independent generation, IgM^+^IgD^+^CD27^+^ B cells were also identified during human fetal development, where one does not expect immune responses to take place (78). Importantly, however, in these early life and disease settings, only a (small) fraction of the IgM^+^IgD^+^CD27^+^ B cells carried somatic IgV gene mutations, and the mutation frequencies in the mutated cells were mostly low (75, 76, 78). Thus, these cells did not show the typical mutation features of adolescent and adult IgM^+^IgD^+^CD27^+^ B cells. Moreover, it seems that there is not a complete absence of GC–like structures in HIGM1 patients (79), and a potential alternative CD40 receptor has been identified (80), so that a GC derivation of the IgM^+^IgD^+^CD27^+^ B cells is still possible.

Regarding an antigen–independent SHM during early B–cell development, it has also been reported that human transitional B cells (a developmental stage between immature B cells in the bone marrow and mature naive B cells) express low levels of AID (81), and that SHM can be induced in vitro in such B cells through toll–like receptor 9 (TLR9) ligation (82). However, the very low levels of mutations showed a highly untypical pattern, with an accumulation mainly in the framework regions of the V region genes and a restriction to some IGHV subgroups (82). Thus, it is highly unlikely that this is the derivation of the mutations in IgM^+^IgD^+^CD27^+^ B cells. Moreover, there is indication that those immature B cells that express AID are destined to undergo apoptosis (83), further arguing against the idea that this is a pathway to establish a major pool of B cells with mutated IgV genes.

A second scenario for the origin of somatically mutated IgM^+^IgD^+^CD27^+^ B cells is their generation and SHM in TI immune responses. This idea was originally based on the observation that splenectomized and asplenic patients show a specific strong reduction of IgM^+^IgD^+^CD27^+^ B cells, and a predisposition to pneumococcal infections as well as impaired responses to polysaccharide vaccines, which are both thought to be mainly mediated by TI immune responses (61, 84). This led to the concept that IgM^+^IgD^+^CD27^+^ B cells are generated and undergo SHM in TI immune responses, and hence represent TI memory B cells (84). However, according to other studies, removal of the spleen also substantially diminishes frequencies of class–switched memory B cells in the peripheral blood (85–88). It is also an unresolved issue where such TI immune responses accompanied by accumulation of substantial loads of somatic mutations in IgV genes take place (see **Box 1**). A further major caveat in the interpretation of immune responses to TI antigens in humans relates to their potential immune history. In a detailed study of the humoral immune response to a polysaccharide vaccine it was shown that the IgM^+^IgD^+^CD27^+^ B cells that showed a major contribution to this TI stimulation were indeed pre–diversified and had acquired a high load of somatic IgV gene mutations in earlier immune responses, likely against gut–resident bacteria (94). These prior immune responses were likely TD immune responses involving GC reactions, as they also gave rise to numerous clonally related class–switched memory B cells (94). Indications for the reactivation of IgM^+^IgD^+^CD27^+^ memory B cells already present at the time of TI immunization was also provided in other studies (95, 96). Therefore, the frequent responsiveness of IgM^+^IgD^+^CD27^+^ B cells to pure polysaccharide antigens does not establish that these cells were generated in a TI response with SHM taking place outside of GC. It may well be that these cells were generated in earlier immune reactions in which polysaccharides as components of bacteria elicited TD immune responses.

Finally, a GC–independent SHM in the generation of mutated IgM^+^IgD^+^CD27^+^ B cells was also proposed based on the observation that patients with a deficiency in the inducible costimulator (*ICOS*) gene lacked class–switched memory B cells but showed normally mutated IgM^+^IgD^+^CD27^+^ B cells (97). However, small GC structures were detected in a lymph node available from one patient, and it is known from studies in the mouse that ICOS plays an important role in the induction of CSR (98). Thus, it may well be that the rudimentary GC in ICOS–deficient patients give rise to IgV gene mutated IgM^+^IgD^+^CD27^+^ memory B cells, but fail to generate class–switched memory B cells because of impaired class–switching.

## Origin and characteristics of human IgM–expressing memory B cells

In the initial description of specific human peripheral blood IgM^+^ B–cell subsets with somatically mutated IgV genes and a proposed origin from GC reactions, two subsets were distinguished, namely IgM^+^IgD^+^CD27^+^ and IgM^+^IgD^low/–^CD27^+^ (IgM–only) B cells (19, 99). The observation that HIGM1 patients with a germline deficiency of the *CD40L* gene have IgM^+^IgD^+^CD27^+^ but practically no IgM–only B cells was later taken as an argument that these are indeed distinct subsets with distinct origins (75). However, our own transcriptomic analysis of such cells revealed IgD as the only significantly differentially expressed gene (29), and cells of these subsets show a very similar BCR repertoire and often common B–cell clones (25). As IgM–only B cells have slightly higher IgV gene mutation frequencies than IgM^+^IgD^+^CD27^+^ B cells (25), it is likely that IgM–only B cells typically develop later in the GC response (i.e., after more rounds of proliferation and mutation) than the IgM^+^IgD^+^CD27^+^ B cells and that this is associated with a preferential downregulation of IgD. In disease situation such as the HIGM1 syndrome with an impaired GC reaction, the few memory B cells that are generated may not reach the differentiation stage when IgD is typically downregulated. As there is thus indication that the two types of IgM–expressing CD27^+^ memory B cells are largely identical, and as most studies did not distinguish between the large population of IgM^+^IgD^+^CD27^+^ and the rare IgM–only B cells, we focus the following discussion on IgM^+^IgD^+^CD27^+^ cells, but typically mean to include the IgM–only B cells.

Considering the ongoing debate about the origin and identity of IgM^+^(IgD^+^)CD27^+^ B cells, we discuss the characteristic features of these cells with a focus on the question whether these features argue for a derivation of these cells from TD immune responses and their memory B–cell identity.

First, the IgV mutation frequency of IgM^+^IgD^+^CD27^+^ B cells is very low in newborns and slowly accumulates with increasing age, as is also typical for classical class–switched memory B cells and as is expected for B cells that are generated in response to infections and vaccinations. Please note that the frequency of IgM^+^IgD^+^CD27^+^ B cells does typically not change with age, in contrast to class-switched memory B cells (**Table 1**) (8, 18, 24, 64, 100). One potential explanation for this is that very early in life GC reactions do not yet function optimally (e.g., because of an immaturity of TFH cells), and that CSR is less efficient, so that GC B cells preferentially differentiate into memory B cells without undergoing CSR.

Second, IgM^+^IgD^+^CD27^+^ B cells are long–lived cells, with a similar longevity as class–switched memory B cells (101), and persistence of IgM memory B–cell clones over years has been observed (102–104). This is a further hallmark of memory B cells. In some specific settings, the frequency of antigen–specific IgM^+^IgD^+^CD27^+^ B cells became reduced in peripheral blood faster than that of IgG^+^ memory B cells (105). However, this may be a specific feature of that particular immune response, and may involve a preferential homing of IgM^+^IgD^+^CD27^+^ B cells with time in secondary lymphoid organs, so that their frequency in peripheral blood is lowered, and/or a further differentiation of a fraction of IgM memory B cells into IgG memory B cells though CSR.

Third, transcriptomic analyses revealed that IgM^+^IgD^+^CD27^+^ B cells have a global gene expression pattern closely related to that of class–switched memory B cells, and clearly distinct from that of naive B cells (29, 31, 33). This similarity is also evident at the protein level, because IgM^+^IgD^+^CD27^+^ and class–switched B cells share a higher expression of several typical B–cell differentiation markers in comparison to naive B cells, including CD24, CD45RB (clone MEM–55), CD80, CD180, and TACI (38, 106), whereas the naive B–cell marker CD23 is neither expressed by IgM^+^IgD^+^CD27^+^ B cells nor by class–switched B cells (107). A further feature where IgM^+^IgD^+^CD27^+^ B cells show a higher global similarity to class–switched memory B cells than to naive B cells is the DNA methylation pattern (108). Hence, also at the epigenetic level, IgM^+^IgD^+^CD27^+^ B cells are similar to classical IgG^+^ memory B cells and substantially more different to pre–GC naive B cells.

Fourth, in vitro stimulation studies revealed that IgM^+^IgD^+^CD27^+^ B cells have the propensity to reacquire GC B–cell features, whereas IgG^+^ memory B cells preferentially differentiate into plasma cells upon restimulation (25, 29). This is reminiscent of the situation in mice, where transfer experiments revealed that GC–derived IgM^+^ memory B cells upon immunization establish new GC in recipient mice, whereas IgG^+^ memory B cells mainly differentiated into plasma cells (109, 110). These observations further support a link of human IgM^+^IgD^+^CD27^+^ B cells to the GC reaction.

Fifth, IgM^+^IgD^+^CD27^+^ and class–switched B cells are in vitro in numerous stimulation conditions easier to activate than naive B cells, and hence share this further hallmark of memory B cells with class–switched memory B cells (29–37).

Sixth, about 20% of IgM^+^IgD^+^CD27^+^ B cells and 30% of IgG memory B cells of human adults carry somatic mutations in the 5’ region of the *BCL6* gene (60). *BCL6* is an off–target for the SHM process and acquires mutations in about 30% of GC B cells, at a frequency about one fifties of the IGHV gene mutation frequency of the same cells (111). As somatic hypermutation is strictly dependent on active transcription of its target genes (112–114), and as BCL6 is only transcribed at substantial levels in GC B cells (115, 116), this argues for a GC derivation of IgM^+^IgD^+^CD27^+^ B cells. The lower frequency of *BCL6*–mutated IgM^+^IgD^+^CD27^+^ than IgG^+^ B cells is to be expected, because the non–class–switched cells also have a similarly lower IGHV gene mutation frequency, likely due to their typically earlier exit from the GC (19). Although this genetic feature of IgM^+^IgD^+^CD27^+^ B cells cannot clarify whether all of these cells are GC–experienced, the frequency of *BCL6*–mutated IgM^+^IgD^+^CD27^+^ B cells nevertheless argues that at least a large fraction of these cells derives from GC B cells.

Seventh, IgM^+^IgD^+^CD27^+^ and class–switched memory B cells show further similarities for two features of their BCR structure and repertoire: The IGHV region genes of class–switched and IgM^+^IgD^+^CD27^+^ B cells have on average shorter CDRIII lengths than naive B cells (23, 38, 65, 117–119), and both B–cell subsets show a counterselection of usage of the *IGHV4–34* gene (23, 38, 65, 119). As IGHV4–34 is inherently autoreactive (120), and as also BCRs with long CDRIII tend to be poly–/autoreactive (121), it is thought that the counterselection against such potentially dangerous features occurs during the GC reaction and is hence a feature of post–GC memory B cells (38, 120, 121). However, in several studies, the overall BCR repertoire in terms of IGHV gene usage of IgM^+^IgD^+^CD27^+^, IgM–only and class–switched memory B cells showed relatively little overlap, and this was taken as an indication that these subsets have a distinct origin (27, 28, 117). A possible explanation for this can be that in many immune responses, preferentially either class–switched or IgM memory B cells are generated, so that the overall repertoires appear distinct, even though both subsets derive from GC reactions. Similarly, the larger number of distinct clones among IgM^+^IgD^+^CD27^+^ B cells compared to class–switched memory B cells in young children (85) may reflect that there is less efficient CSR in GC in the first two years of life, so that a polyclonal pool of non–class–switched memory B cells is already established when IgG^+^ memory B cells are still few and derive from fewer clones.

Eighth, a further, and perhaps the key genetic characteristic feature of GC–derived memory B cells is the presence of somatic mutations in the rearranged IgV genes of nearly all of these cells. This is also true for IgM^+^IgD^+^CD27^+^ B cells, which show an average IGHV mutation frequency of 4–5% in adults (19, 23, 25). As the GC is the only known structure where substantial levels of SHM are acquired in humans (**Box 1**), this strongly argues for a GC derivation of most if not all IgM^+^IgD^+^CD27^+^ B cells, at least in adults.

Nineth, and related to the prior feature, we and others identified numerous large memory B–cell clones which were composed of IgM^+^IgD^+^CD27^+^ and class–switched B cells (25, 60, 94, 100, 103, 104, 117, 122). The hierarchical trees generated from the mutated IgV gene sequences frequently showed an intermingling of IgM– and IgG–expressing B cells, which is a strong argument that these clone members derive from a common mutating GC B–cell clone. Notably, IgM^+^IgD^+^CD27^+^ B cells often were closer to the root of the trees than class–switched memory B cells (25, 60). This fits very well to the observation that the IgV genes of IgM^+^IgD^+^CD27^+^ B cells are on average less mutated than class–switched memory B cells and explains this by indicating that the IgM–expressing memory B cells typically exit the GC after fewer rounds of proliferation and mutation than IgG^+^ and IgA^+^ B cells, and have hence acquired less mutations. Clonal relationships between IgM– and IgG–expressing B cells were in some studies only rarely or not at all observed (27, 28). However, one has to consider that in most studies only a relatively small number of B cells was studied, considering the size of the overall B–cell pool of an individual. Also in our own studies, there was a clear tendency that the chances to identify common clone members among IgM^+^ and class–switched memory B cells was higher when the sample size or the overall size of a clone was larger (25). In further support of a GC derivation of IgM^+^IgD^+^CD27^+^ B cells, in a study that included deep sequencing of IgV genes from intestinal GC and from memory B–cells, common clones of intestinal GC B cells and IgM^+^IgD^+^CD27^+^ B cells with intermingling mutation patterns were identified (123).

Box 1 **Considerations for SHM outside GC or in TI GC responses**

Considering the hypothesis of SHM in human B cells taking place outside of the GC, there are indeed AID–expressing B cells detectable outside of GC in secondary lymphoid organs (89, 90). However, these are singular cells, and it is likely that these are B cells induced to undergo CSR, which is also dependent on AID expression. It cannot be excluded that some AID–expressing B cells outside GC acquire IgV gene point mutations. However, one has to consider that IgM^+^IgD^+^CD27^+^ B cells in adolescents and adults carry a substantial level of somatic mutations in the IGH and IGL V genes (4% on average), and that most mutations are disadvantageous. This therefore argues that a stepwise process of proliferation, somatic hypermutation and selection as in the GC is needed to generate memory B cells with often more than ten point mutations per IgV gene and a functional and antigen–specific BCR. Importantly, clusters of AID–expressing B cells were so far not detected outside of GC in humans (89–91), so that it remains enigmatic where and how well–mutated IgM^+^IgD^+^CD27^+^ B cells would obtain their IgV gene mutations outside GC.

A further source of IgM^+^ memory B cells could potentially be TI immune responses that involve GC–like structures. There are indeed reports that immunizations of mice with TI antigens can induce GC–like structure, and that some of these GC B cells can develop into memory B cells (92). However, these GC–like structures developing in the absence of T cells are short–lived and vanish after a few days (92, 93). Therefore, it is highly unlikely that such structures can give rise to well mutated memory B cells, such as human IgM^+^IgD^+^CD27^+^ B cells. Moreover, it seems that GC without any TFH cells have not been identified in healthy humans.

Tenth, in a number of specific infections and immunizations, antigen–specific IgM^+^IgD^+^CD27^+^ memory B cells were identified besides class–switched memory B cells, and sometimes they were even the dominant memory B–cell subset. This includes immune responses against rotavirus (124, 125), hepatitis B virus (126), papilloma virus (127), polyomavirus (128), Rhesus D blood group antigen (129), SARS–CoV–2 (102), tetanus toxoid (129, 130), *Streptococcus pneumoniae* (95), and *Plasmodium falciparum* (131). This clearly shows that in these typical TD immune responses, the memory B–cell compartment also encompasses IgM^+^ memory B cells.

While the multiple characteristic features of IgM^+^IgD^+^CD27^+^ B cells discussed above in our view strongly argues for their GC derivation and memory B–cell identity, it remains unclear how to reconcile this with the indications for a GC–independent generation of these cells. Considering that most studies supporting the idea of a GC– and/or T cell–independent origin were focused on such B cells in children, whereas most studies supporting a GC–derivation of IgM^+^IgD^+^CD27^+^ B cells were performed with B cells from human adults, one may speculate that there might be different developmental pathways for these B cells active in early childhood versus adulthood, and that IgM^+^IgD^+^CD27^+^ B cells are not a homogenous population but may indeed encompass distinct subsets. A direct analysis of this idea was recently published by Grimsholm and colleagues (65). They distinguished two subsets of IgM^+^IgD^+^CD27^+^ B cells based on the expression level of CD27 (65). In very young children a CD27^dull^IgM^+^IgD^+^ B–cell population with lowly mutated IgV genes dominated, whereas with increasing age, CD27^bright^IgM^+^IgD^+^ B cells with higher mutation loads predominated (61, 65). In patients with immunodeficiencies that are expected to impair GC responses (e.g., HIGM1), CD27^bright^IgM^+^IgD^+^ B cells were missing, but CD27^dull^IgM^+^IgD^+^ B cells were present, leading the authors to conclude that the CD27^dull^IgM^+^IgD^+^ B cells can be generated in a GC independent way, whereas CD27^bright^IgM^+^IgD^+^ B cells are post–GC memory B cells (65).

Taken together, there is now strong evidence that the vast majority of human peripheral blood IgM^+^IgD^+^CD27^+^ B cells in adults are post–GC memory B cells, but in younger children a considerable subset of these cells may derived from a so far poorly understood distinct developmental pathway, which may not involve conventional GC reactions.

## Splenic marginal zone B cells

The human spleen is the largest secondary organ of the immune system and has important functions in blood filtration, iron metabolism, and immune defense, and is a major reservoir of lymphocytes (132). The splenic marginal zone (sMZ) is a unique area of reduced blood–flow that allows close contact of blood–borne antigens or pathogens with specialized immune cells (133). Thereby, the cells of the sMZ provide immunosurveillance of the peripheral blood.

In order to fulfill their important function in the first line of defense against pathogens invading the bloodstream, sMZ B cells require additional signals from bystander cells in their microenvironment (134, 135). Multiple studies have provided insight into the crosstalk among human sMZ B cells and innate immune cells, such as dendritic cells, natural killer cells, neutrophils, and several types of stromal cells (136–142). These cells provide strong signals via BAFF, APRIL, and DLL1 (141, 143, 144). Human sMZ B cells seem to be less dependent on canonical T–cell help (e.g., through CD40L) and are highly dependent on microbial signals for their recruitment or homeostasis (75, 85, 145). In addition, the literature supports the view that gut microbiota–derived antigens might play a role in sMZ B–cell homeostasis in humans (123, 146–149). These observations support a role of sMZ B cells in TI immune responses, but with substantial help from various innate immune cells. It has been reported that interaction of sMZ B cells with neutrophils in vitro induces SHM in their IgV genes (137). However, the increased somatic mutation load seen in the BCRs may simply represent preferential survival and/or outgrowth of (more highly) mutated B cells in the prolonged cultures.

**Figure 3:**
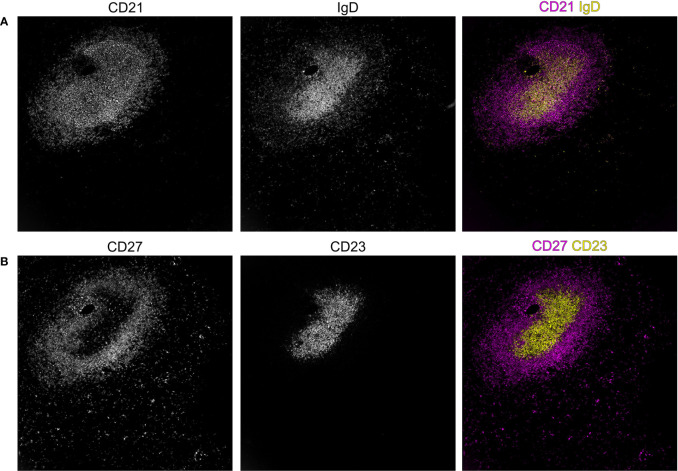
Spatial distribution of B-cell subsets in the splenic white pulp of an adult human individual. In secondary lymphoid organs, such as the spleen, B–cell subsets segregate to different compartments as can be shown by the expression of the markers IgD, CD21, CD23, and CD27. The staining of CD21 shows the B–cell follicle, revealing a compartment of CD21^high^ follicular dendritic cells (FDC), a population of fibroblasts that supports adaptive immunity in the germinal center reaction by its unique capability to acquire and present antigen to B cells in its native form. In addition to the dense area of CD21^high^ FDCs, a more superficial population of CD21 expressing cells is visible in **(A)**, highlighting the B cells of the sMZ, which also co-express the memory B–cell marker CD27 **(B)** in adults. In contrast, **(A)** IgD– and **(B)** CD23–expressing cells are confined to the center of the follicular region. Stainings were performed with the MACSima platform (Miltenyi Biotech, Bergisch Gladbach, German). The dimensions of the images are 750 x 750 µm.

The complex crosstalk in the human sMZ is thought to recruit circulating B cells and to induce a primed state (135). The reprogramming of circulating B cells into the sMZ B–cell compartment does not favor one specific B–cell subset, but naive, IgM memory as well as class–switched memory B cells are susceptible to DLL1–NOTCH2–dependent in vitro induction of the sMZ B–cell phenotype and gene expression profile (100, 150). However, in adults, the vast majority of sMZ B cells harbor mutated IgV genes and already in young children the sMZ precursor population expresses CD45RB^MEM55^, which is a memory B cell marker (150, 151). This indicates that memory B cells have an intrinsic advantage over naive B cells in homing to the sMZ and undergoing reprogramming to sMZ B cells (**Figure 3**).

Human sMZ B cells were defined as the human counterpart of murine sMZ B cells by their homologous localization in the microanatomical niche at the interface of splenic B–cell follicles and the red pulp (152). Murine sMZ B cells and human sMZ B cells furthermore share functional propensities and express similar phenotypes, e.g., high expression of CD21 and IgM, but low expression of IgD (136, 138, 153). Furthermore, signaling through the NOTCH2 receptor seems to be essential for the generation of sMZ B cells not only in the mouse but likely also in humans (100, 154). However, there are also significant interspecies differences between humans and rodents in regard to the organization of the white pulp, the structure of the sMZ (133, 155), the phenotype and IgV gene mutation status of sMZ B cells in adult individuals (135, 153, 156), as well as their functional capacities, which caused dispute about the identity and memory character of human sMZ B cells (85, 157). A more detailed comparison of human and murine sMZ B cells was already covered in multiple publications (100, 134, 135, 155, 158).

Importantly, rodent sMZ B cells mostly carry unmutated IgV genes (159), although there is indication that these cells also encompass a subset of memory B cells from TD immune responses (160). In distinction to this, in humans, as discussed in the previous chapter in the case of blood-derived IgM^+^IgD^+^CD27^+^ B cells, responsiveness to TD antigens, CD27-expression and IgV-gene and *BCL6* mutations indicate a GC-derivation of human sMZ B cells (64, 85, 100, 153, 156, 161). BCR repertoire deep sequencing analyses point to the existence of a body–wide clonal network, which interconnects sMZ B cells with memory B–cell clone members in distant lymphoid organs and supports the view that human sMZ B cells can undergo subsequent GC reactions and recirculate to distant organs via the blood stream (88, 162–164). It was also observed that many circulating memory B cells are clonally related to sMZ B cells, supporting the idea of a systematic memory B–cell archive in the spleen (134).

Besides the indications for a derivation of at least a large fraction of sMZ B cells from TD immune responses, there are also indications for a generation of sMZ B cells in TI immune responses. Pioneering studies have established that the susceptibility to infections with encapsulated bacteria upon splenectomy is a consequence of the concomitant loss of sMZ B cells (84, 87) and their specialized microanatomical niche, which is strategically placed at the border of the splenic immune compartment and the circulatory compartment. SMZ B cells show unique response kinetics upon stimulation and their frontline localization allows sMZ B cells and sMZ–resident phagocytes to bind and remove circulating pathogens from the bloodstream through the induction of TI antibody responses (38, 75, 135, 137, 165).

Intriguingly, not only splenectomy renders individuals more susceptible to bacterial infections, which are thought to often cause TI immune responses, but also age is a critical determinant of splenic immune function: Newborn, young children, and seniors are at increased risk of suffering from severe infections with encapsulated bacteria (166, 167). It was suggested that the vulnerability in young individuals is a result of incomplete maturation of the sMZ B–cell compartment (168, 169). Our own results showed that sMZ B cells are already detectable only two weeks after birth and show equivalent response dynamics as adult–derived sMZ B cells, however, lacking IgV gene mutations and a compartment of class–switched sMZ B cells (64, 100). This indicates that a large fraction of these sMZ B cells in very young individuals have only limited or no GC experience.

There were no significant differences regarding CDRIII length distribution, IgV gene usage, and IgV gene mutation load when comparing the BCR repertoire of paired sMZ B cells to non–sMZ B cells derived from spleen and blood of the same individual. This finding indicates that the sampling process of splenic B cells into the sMZ compartment is largely random (64). In addition, the spleen seems to induce a distinct phenotype among memory B cells, which differs from the equivalent B–cell subsets in blood and other secondary lymphoid organs, e.g., in higher expression of CD21, CCR7, and CD180 by sMZ B cells, or a higher fraction of CD69– and CD45RB–expressing cells (123, 170). These phenotypical adaptations of splenic B cells might contribute to immune protection against blood–borne pathogens.

In seniors, splenic naive B cells are virtually absent and the fraction of class–switched sMZ B cells that harbor highly mutated IgV genes is massively increased. Moreover, few, extremely large, mostly class–switched B–cell clones dominate the splenic BCR repertoire at the expense of smaller clones in the elderly (100). This skewed composition of the splenic B–cell pool in elderly individuals possibly contributes to the diminished efficiency of immune responses against newly encountered pathogens, vaccinations, and polysaccharide antigens in old age (166, 167).

Taken together, the sMZ B–cell compartment seems to change substantially during the life of humans and there is ample evidence that these B cells may either derive from TI or TD immune responses, and may also have functions in both types of immune responses. This is indeed important to distinguish, because it is well known that GC–derived memory B cells may be reactivated without T cell help (94, 96), so an engagement of sMZ in TI immune responses as such does not argue that the cells were not originally generated in GC reactions of TD responses. A possible solution to the seemingly contradictory origins of sMZ B cells could be that the cells are not a homogenous population, but that they include distinct B–cell subsets with distinct origins. There are indeed indications for a heterogeneity of these cells (64, 100, 123). A mass cytometry and single cell RNA–sequencing study identified two subsets of human sMZ B cells, one with unmutated or lowly mutated IgV genes and further characteristics of a GC–independent generation (termed MZB–2), and the other with well mutated IgV genes and further memory B–cell features (e.g., higher CD27 expression; MZB–1) (147). The CD27^low^ MZB–2 subset was a minor subset in adults. Notably, the MZB–1 cells were frequently clonally related to IgM–only B cells (147), a bona–fide post–GC memory B–cell population, as discussed above. The identification of a specific human sMZ precursor population that is frequent early in life but diminishes with increasing age (150), fits to the concept that in young children the sMZ is mainly populated by GC–inexperienced (but nevertheless perhaps often memory) B cells that are also mainly involved in TI immune responses. With increasing age these cells are continuously replaced by post–GC, in young children mostly IgM–expressing, but in older children and adolescents also IgG^+^ memory B cells that can be engaged in both TI and TD immune responses.

## Concluding remarks

Human IgM^+^IgD^+^CD27^+^ and IgM–only B cells together account for about 20% of the B-cell pool in human adults, so that it is clearly highly relevant to know the origin and function of these cells for a comprehensive understanding of humoral immunity in humans. After more than 20 years of controversial discussions about the origin and function of these IgM–expressing B cells with somatically mutated IgV genes (19, 75, 99), now a scenario is developing which may resolve the controversies and provide an integrative view on the numerous studies on this topic. Based on studies either focused on B cells in the peripheral blood or those in the spleen, it seems that early in life, some IgM^+^IgD^+^ B cells may undergo a low level of SHM outside of GC reactions and TD immune responses. While independent studies of fetal or early life B cells or B cells from patients with particular immunodeficiencies and single cell transcriptomic studies of splenic B cells support this concept (65, 75, 76, 78, 147, 150), it is still unresolved where in the human body SHM takes place outside of GC, and to what extent these early life lowly IgV mutated IgM^+^IgD^+^CD27^+^ B cells derive from either an antigen–independent primary diversification process or from TI immune responses. Soon after birth, when children are exposed to a myriad of foreign antigens driving TD immune responses, a population of IgM^+^IgD^+^CD27^+^ B cells emerges with all hallmark features of post–GC TD memory B cells: longevity, well mutated IgV genes, pathogen specificity, fast and easy response kinetics, and expanded B–cell clones. In the adult, these post–GC IgM^+^IgD^+^CD27^+^ memory B cells represent the vast majority of the IgM^+^IgD^+^CD27^+^ B cells in peripheral blood and the sMZ (19, 65, 75, 99, 147), so the contributions of the early life non–GC derived IgM^+^IgD^+^CD27^+^ B cells become negligible. Direct evidence for two distinct subsets of IgM^+^IgD^+^CD27^+^ B cells both in peripheral blood and in the spleen, with one showing indications for a GC independent generation and dominating early in life, and the other with key post–GC memory B–cell features dominating in adulthood, strongly support this scenario (64, 65, 147).

Regarding the numerous features brought forward earlier in the review to argue for a GC derivation of IgM^+^IgD^+^CD27^+^ B cells in adults, we consider some arguments as particularly strong. This includes the detection of shared clones with intermingling IgV gene mutation patterns with classical class–switched memory B cells (25, 60, 94, 100, 103, 117, 122), the presence of *BCL6* mutations in a considerable fraction of these cells (60, 161), and the existence of long–lived IgM^+^IgD^+^CD27^+^ B cells among pathogen–specific memory B cells for numerous viral and bacterial species that elicit TD humoral immune responses (95, 102, 124–131). Other features of these cells in common with class–switched memory B cells, such as the transcriptomes, specific BCR repertoire features, surface marker expression patterns, and in vitro reactivities, may be primarily regarded as only correlative and might also be features of the proposed memory B cells derived from TI responses. However, in our view, the multitude of common characteristic of IgM^+^IgD^+^CD27^+^ B cells with GC–derived IgG^+^ and IgA^+^ memory B cells indeed strongly supports a common origin of these cells from GC reactions. The advantage for the immune system to have distinct subsets of memory B cells is obvious: The more highly IgV mutated class–switched memory B cells can quickly differentiate into plasma cells when encountering again the specific pathogens that led to their generation for a fast increase in specific antibody production. IgM^+^IgD^+^CD27^+^ B cells can also undergo such differentiation and can become IgM–secreting plasma cells, which conveys highly efficient complement fixation. Importantly, IgM^+^IgD^+^CD27^+^ B cells additionally have the propensity upon re–encounter of the same or a related antigen to again enter GC reactions, to adopt the BCR specificity to a potentially altered antigen through new rounds of SHM and selection (2). These IgM–expressing memory B cells also have the flexibility to switch to a particular IgG or IgA class, depending on what is needed for the secondary immune response. They are thus a particularly flexible arm of our immunological memory.

## Author contributions

BB: Conceptualization, Writing – original draft, Writing – review & editing. AK: Conceptualization, Writing – original draft, Writing – review & editing. RK: Conceptualization, Writing – original draft, Writing – review & editing.
